# MicroRNA-210-3p Targets RGMA to Enhance the Angiogenic Functions of Endothelial Progenitor Cells Under Hypoxic Conditions

**DOI:** 10.3389/fncel.2019.00223

**Published:** 2019-05-21

**Authors:** Wen-Jing Lu, Huai-Bin Liang, Yong-Fang Li, Xuan-Qiang Tu, Ji-Rong He, Kai-Qi Ding, Guo-Yuan Yang, Xiao-Yu Xin, Li-Li Zeng

**Affiliations:** ^1^Department of Neurology, Ruijin Hospital, School of Medicine, Shanghai Jiao Tong University, Shanghai, China; ^2^Department of Rehabilitation Medicine, Huashan Hospital, Fudan University, Shanghai, China

**Keywords:** angiogenesis, endothelial progenitor cells, oxygen-glucose deprivation injury, microRNA-210-3p, repulsive guidance molecule A

## Abstract

Endothelial progenitor cells (EPCs) are multipotential stem cells considered to have immense clinical value for revascularization. However, the clinical application of EPCs has been hampered by their clinical potency in ischemic anoxic environments. This study aimed to explore the effect of microRNA-210 (miR-210) on EPCs under oxygen-glucose deprivation (OGD) conditions. We generated a model of EPCs cultured under OGD conditions to simulate ischemia and explore the expression of miR-210 *in vitro*. With longer exposure to hypoxia, we found that miR-210-3p expression was highly upregulated in OGD groups compared to that in controls from 4 to 24 h, but not miR-210-5p. We then transfected a miR-210-3p mimic and inhibitor into EPCs, and after 24 h, we exposed them to OGD conditions for 4 h to simulate ischemia. We detected miR-210 by real-time polymerase chain reaction (RT-PCR) and tested the proliferation, migration, and tube formation of normal EPCs and OGD-treated EPCs by CCK-8, transwell chamber, and Matrigel assays, respectively. The direct targets of miR-210-3p were predicted using miRWalk. Compared to that in normal EPCs, higher miR-210-3p expression was found in OGD-treated EPCs (*p* < 0.05). Moreover, upregulation of miR-210-3p was found to promote proliferation, migration, and tube formation in EPCs under normal and OGD conditions (*p* < 0.05), whereas down-regulation inhibited these abilities in OGD-treated EPCs (*p* < 0.05). Repulsive guidance molecule A (RGMA), a negative regulator of angiogenesis, was predicted to be a target of miR-210-3p. Accordingly, upregulation of miR-210-3p was found to inhibit its expression at the protein level in OGD-treated EPCs, whereas downregulation of miR-210-3p inhibited its expression (*p* < 0.05). A dual-luciferase reporter system confirmed that RGMA is a direct target of miR-210-3p. MicroRNA-210-3p overexpression enhances the angiogenic properties of OGD-treated EPCs by inhibiting RGMA.

## Introduction

Characterized by high mortality, morbidity, and disability, ischemic stroke causes immense health and economic burdens to families and society (Benjamin et al., 2017). However, the pathogenesis of stroke remains unclear and there are currently no effective treatments.

Endothelial progenitor cells (EPCs) are multipotential stem cells considered to have immense clinical value for revascularization. Previous research has demonstrated that EPCs can differentiate into mature endothelial cells (ECs) to take part in angiogenesis and vasculogenesis after cerebral ischemic injury (Chong et al., 2016). They have also been shown to participate in the repair of dysfunctional endothelia and to suppress endothelial injury through their direct incorporation into newly forming vessels or the secretion of pro-angiogenic growth factors or cytokines (Asahara et al., 2011). However, the clinical application of EPCs has been hampered by the limited numbers of surviving cells and their clinical potency in ischemic anoxic environments, in addition to the controversies surrounding their identities and functions (Zhao et al., 2013). Our study began with the key point of improving the ability of EPCs to resist hypoxia and ischemia.

As a class of conserved, single-stranded, endogenous, non-coding RNA, microRNAs (miRNAs) exert physiological effects mainly by negatively regulating gene expression at the post-transcriptional level. Further, they have been widely researched in both experimental and clinical settings. Usually, one miRNA can target more than one mRNA and one mRNA is targeted by more than one miRNA (Lim et al., 2005). This phenomenon results in the formation of an intricate regulatory network that plays important roles in biological processes such as cell proliferation, differentiation, apoptosis, early development, immunity, hematopoiesis and longevity, and angiogenesis, among others (Xu et al., 2003; Calin et al., 2004; Chen, 2004; Wang et al., 2009; de Rie et al., 2017). MicroRNA-210 (miR-210), the key hypoxia-related miRNA, was found to be upregulated in many different types of cells under hypoxic conditions. Studies revealed that this miRNA is involved in angiogenesis, regulating the cell cycle, differentiation, deoxyribonucleic acid repair, and apoptosis by targeting Ephrin-A3, E2F3, NPTX1, RAD52, ACVR1B, MNT, and CASP8AP2, among others (Chan and Loscalzo, 2010). Moreover, miR-210 improves cardiac function in a murine model of myocardial infarction by inhibiting apoptosis and enhancing angiogenesis (Hu et al., 2010). We have previously performed the peripheral blood miRNAs expression profiling in patients with acute cerebral ischemia (Zeng et al., 2016), and miR-210 is one of 10 significant differentially expressed miRNAs. Our previous studies also demonstrated that it is upregulated during the acute stage following cerebral ischemia in transient middle cerebral occlusion (tMCAO) mice (Zeng et al., 2016). Further, its overexpression was found to promote angiogenesis and neurogenesis and improve the prognosis of mice with cerebral ischemia by upregulating vascular endothelial growth factor (VEGF) and brain-derived neurotrophic factor (BDNF) levels (Zeng et al., 2014). MiR-210 has robust anti-hypoxia ability (Chan and Loscalzo, 2010), and it has been reported that miR-210 could enhance the hypoxia tolerance of bone marrow mesenchymal stem cells (MSCs) (Chang et al., 2013). Recent research has shown loading miR-210 in EPCs derived exosomes could boost the beneficial effects on hypoxia/reoxygenation injured human ECs (Ma et al., 2018). However, the function of miR-210 on EPCs was not previously known. Therefore, we hypothesized that the function of EPCs under ischemic conditions might be improved by miR-210 overexpression. We considered that the combination of miR-210 and EPCs could be a more effective therapy for treating stroke.

In this study, we used an *in vitro* cell model with oxygen-glucose deprivation (OGD) injury to simulate an ischemic environment. We investigated the possible functions of miR-210 in EPC under the hypoxic condition and further explored the potential underlying molecular mechanism in controlling cellular behavior.

## Materials and Methods

### Cell Culture and OGD Treatment

Endothelial progenitor cells were extracted from the umbilical cord blood of the healthy parturient women. Umbilical cord blood was provided by the International Peace Maternity and Child Health Hospital, School of Medicine, Shanghai Jiao Tong University, Shanghai, China. And all participants were informed about the study protocol and given written informed consent according to the Declaration of Helsinki principles. The study protocol was approved by the Ethics Committee of Ruijin Hospital, Shanghai Jiao Tong University School of Medicine. Cells were cultured in Endothelial Growth Medium-2 (EGM-2) (Lonza, United States) at 37 ± 1°C with 5% CO_2_, 90 ± 2% humidity in 10-cm^2^ culture dishes. To mimic ischemia conditions *in vitro*, the culture medium was replaced and washed with phosphate buffered saline (PBS) (HyClone, United States), and cells were placed in an airtight experimental hypoxia chamber containing a gas mixture comprising 95% N_2_ and 5% CO_2_ for 4, 12, or 24 h. EPCs cultured with normal oxygen levels served as controls. All cells were collected for total RNA extraction using TRIzol (Invitrogen, United States). EPCs used in all experiments were fourth to the sixth generation.

### Immunocytochemistry

Endothelial progenitor cells were washed with PBS (HyClone, United States), and then fixed in 4% paraformaldehyde (PFA) for 10 min. EPCs were then washed with PBS three times after PFA was discarded. Cells were permeabilized with 0.3% Triton-100 (Sigma-Aldrich, United States) for 10 min and blocked with 10% bovine serum albumin (BSA) (Sigma-Aldrich, United States) for 1 h. EPCs were incubated for 20 h at 4°C with primary antibodies in PBS as follows: polyclonal sheep anti-CD31 (1:200; R&D, United States), polyclonal sheep anti-CD34 (1:200; R&D, United States), polyclonal rabbit anti-Von Willebrand Factor (vWF) (1:200; Abcam, United Kingdom), polyclonal goat anti-KDR (1:200; R&D, United States), polyclonal rabbit anti- repulsive guidance molecule A (RGMA) (1:300; Abcam, United Kingdom). The secondary antibodies were Alexa 488/594-conjugated donkey anti-sheep/rabbit/goat (1:1000; Invitrogen, United States) and the nuclei were stained by adding DAPI (1:1000; Invitrogen, United States). All samples were observed with a confocal laser scanning microscope (Leica, Germany).

### Flow Cytometry

Endothelial progenitor cells were digested and centrifugalized when the cell density was 80–90%. Cells were then resuspended in PBS at a concentration of 1 × 10^7^/ml. We used 1 × 10^6^ cells in a 100-μl sample (test). EPCs were incubated with fluorescent antibodies including CD34-PE (1:5; BD Biosciences, United States), CD31-PE (1:5; BD Biosciences, United States), KDR-APC (1:5; BD Biosciences, United States), CD105-APC (1:20; BD Biosciences, United States), and CD133-PE (1:10; Miltenyi Biotec, Germany) in the dark for 30 min on the ice. Without washing, samples were analyzed by flow cytometry.

### Cell Transfection

For miR-210-3p overexpression and suppression, a miR-210-3p mimic (5′-CUGUGCGUGUGACAGCGGCUGA-3′), miR-210-3p inhibitor (5′-UCAGCCGCUGUCACACGCACAG-3′), mimic control (5′-GCGCUCGUGGAGUGCGUGUCAA-3′) and inhibitor control (5′-GCCAGAGCCUGACCGCUACCUA-3′) were purchased from TuoRan (Shanghai, China). According to the manufacturer’s protocol, we transfected mimic or mimic control at a concentration of 50 nM and inhibitor or inhibitor control at a concentration of 200 nM using Lipofectamine 2000 (Invitrogen, United States) in accordance with the manufacturer’s instructions. All related experiments were performed within 24 h after transfection.

### RNA Extraction, Reverse Transcription, and Real-Time PCR

Using the protocol for TRIzol (Invitrogen, United States), RNA extraction was performed. RNA concentration and purity were detected using the NanoDrop1000 spectrophotometer (Thermo Fisher Scientific, United States). Samples with an absorbance ratio at 260–280 nm between 1.8 and 2.0 were adopted. The Starter Kit (EXIQON, Denmark) was used for reverse transcription and real-time quantitative polymerase chain reaction (qPCR) according to the manufacturer’s protocol in the measurement of miR-210. A fast real-time PCR system (7900 HT, ABI) was used for this amplification. The relative expression of miR-210 was normalized to the expression of the internal reference U6 and calculated by the 2^–ΔΔCt^ relative quantification method. As for RGMA (forward primer 5′-TCACCGACCGCTTCCAGACC-3′; reverse primers 3′-CT CCTTCACCAGTTACGACACCTC-5′), the PrimeScript^TM^ RT reagent Kit (Takara, Japan) was used for reverse transcription and real-time qPCR according to the manufacturer’s protocol in the measurement of RGMA. The relative expression of RGMA was normalized to the expression of the internal reference GAPDH (forward primer 5′-GATGGTGAAGGTCGGTGTGA-3′; reverse primers 3′-TGAACTTGCCGTGGGTAGAG-5′) and calculated by the 2^–ΔΔCt^ relative quantification method.

### Proliferation Assay

We used the cell counting kit-8 (CCK-8) (Dojindo, Japan) to examine EPC proliferation. For this, 1 × 10^4^ cells with 100 μl of EGM-2 were inoculated into each well of a 96-well plate and incubated for 24 h. Then, a new medium containing CCK-8 reagent (10:1 v/v) was added to each well. Cells were further incubated at 37°C for 2 h. The absorption at 450 nm was examined using a microplate reader.

### Migration Assay

As a classical *in vitro* model of angiogenesis, migration was assessed in 24-well plates with 8.0-mm pore polycarbonate membranes inserted (Corning, United States). In brief, we added 800 μl of EGM-2 into each well of the 24-well plate, which was followed by inserting chambers with polycarbonate membranes. Then, 2 × 10^4^ cells with 200 μl of EBM-2 were added into each chamber in triplicate. After further incubation for 20 h, cells were fixed with PFA and stained with crystal violet for 10 min. The images of cells that migrated across the membrane were captured using a microscope (Leica, Germany). The tinctorial cells under the membranes were then counted and analyzed.

### Tube Formation Assay

As another classical *in vitro* model of angiogenesis, tube formation assays were carried out in 96-well plates. Approximately 40 μl of Matrigel (BD, United States) was added into each well of a 96-well plate and incubated at 37°C for 30 min for solidification. Then, 1.5 × 10^4^ cells were inoculated into the coated plate in triplicate. After culturing for 8 h at 37°C. After having obtained a series of three-dimensional focal stack images of the microvascular structure via a bright field microscope, we imported the stack images into Intensify3D to enhance and optimize the cell signal (Yayon et al., 2018). Then we used the AngioTool to calculate the tube length as previously reported (Zudaire et al., 2011).

### Western Blotting

Endothelial progenitor cells were lysed and proteins were extracted separately from cells with radioimmunoprecipitation assay buffer (10 mM Tris/HCl, pH 8.0, 150 mM NaCl, 1 mM EDTA, 1% sodium deoxycholate, 1% Triton X-100, 0.1% SDS, and protease inhibitor cocktails) at 4°C and centrifuged at 12000 × *g* for 25 min to remove cell debris. The supernatant was transferred to a new tube and mixed with 5× loading buffer. For the detection of RGMA, samples were electrophoresed with a 10% polyacrylamide gel, and the proteins were electrophoretically transferred to polyvinylidene difluoride membranes. The membranes were blocked with Block Ace (Sangon, China) and incubated with a polyclonal rabbit anti-RGMA antibody (1:1000; Abcam, United Kingdom) and a monoclonal mouse anti-GAPDH antibody (1:1000; Invitrogen, United States) in TBST for 16 h at 4 °C. Antibodies were detected by incubating the samples with an HRP-conjugated secondary antibody (1:5000; Invitrogen, United States) for 1 h at room temperature. The blots were detected using Super Signal West Femto Substrate (Thermo Fisher Scientific, United States) and a LAS-4000 imaging system. For densitometric quantification, the band density of RGMA relative to that of β-actin was quantified by NIH ImageJ software.

### Luciferase Reporter Assays

In this study, the full length of the 3′UTR sequence was cloned (base from 1636 to 3227) to construct the luciferase reporter vectors. The full 3′UTR fragments from *RGMA* containing the predicted miR-210-3p-binding sites were amplified by PCR and subcloned downstream of the luciferase gene in the luciferase vector. The 3′UTR of *RGMA* with or without the mutant sequence was then amplified. For luciferase assays, EPCs were cultured in 24-well plates and co-transfected with 200 ng of firefly luciferase constructs, 4 ng of *Renilla* luciferase plasmid, and 50 nmol/L miR-210-3p mimic using Lipofectamine 2000. *Renilla* luciferase activity was measured using a dual-luciferase reporter assay 48 h after transfection. The results were expressed as relative luciferase activity (firefly luciferase/*Renilla* luciferase).

### Data Analysis and Statistics

All statistical tests were performed using GraphPad Prism7. Data are presented as the mean ± SEM. Three or more replicates were always performed for each experiment in all assays. Statistical analyses were performed by one-way ANOVA followed by Student-Newman-Keuls tests. A probability value of *p* < 0.05 was considered statistically significant.

## Results

### Culture and Identification of EPCs

**FIGURE 1:**
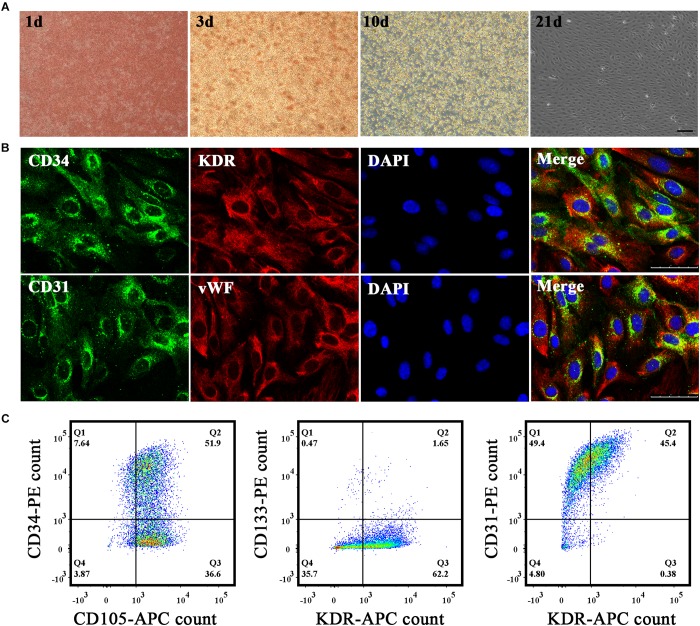
Cultivation and identification of endothelial progenitor cells (EPCs). **(A)** EPCs were extracted from umbilical cord blood and morphological changes in primary EPCs were observed *in vitro* on the first, third, tenth, and twenty-first days successively under a microscope. Bar = 100 μm. **(B)** Immunohistochemistry staining results to identify EPCs. Biomarkers adopted were CD34, CD31, KDR, and vWF. Bar = 50 μm. **(C)** Flow cytometry results to identify EPCs. Biomarkers adopted were CD34, CD105, CD133, KDR, and CD31.

We extracted mononuclear cells from the umbilical cord blood of healthy parturient women using lymphocyte separation liquid. The medium was changed twice per week until primary EPCs were passaged. Primary EPCs grew out at approximately the third week. We could see small, cobblestone-shaped cell clusters and growth and proliferation were highly apparent (Figure 1A). We subjected the fourth generation of EPCs to direct staining (Figure 1B) and under the microscope, we could see that nearly half of EPCs were stained for CD34 (green) and CD31 (green), whereas most of the EPCs were stained for KDR (red) and vWF (red). Flow cytometric analysis (Figure 1C) was used to identify the proportion of EPCs that were stained with endothelial surface antigens (CD105, 88.5%; CD31, 94.8%; KDR, 45.78∼63.85%) and stem cell surface markers (CD34, 59.54%; CD133, 2.12%). Our results thus showed that cultured cells derived from human cord blood were EPCs.

### Upregulation of MiR-210-3p in EPCs Under OGD Conditions

**FIGURE 2:**
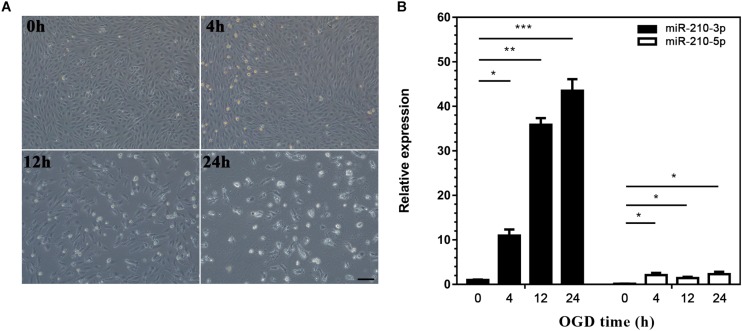
Construction of oxygen and glucose deprivation (OGD) model and expression of microRNA (miR)-210. **(A)** Endothelial progenitor cells (EPCs) were exposed to OGD conditions for 0, 4, 12, and 24 h to simulate ischemia. Bar = 100 μm. **(B)** Real-time PCR was used to detected miR-210 expression in OGD-treated EPCs; *n* = 3 per group. Data are presented as the mean ± SEM, *p < 0.05, **p < 0.01, and ***p < 0.001.

We next generated a model of EPCs cultured under OGD conditions to simulate ischemia and explore the expression of miR-210 *in vitro*. Although the majority of EPCs survived when deprived of oxygen and glucose for 4 h, cell debris could still be observed under the microscope. With longer exposure to hypoxia, EPCs became deformed and disaggregated and died progressively from 4 to 24 h (Figure 2A). Correspondingly, miR-210-3p expression was highly upregulated in OGD groups compared to that in controls, whereas miR-210-5p expression was upregulated in OGD groups compared to expression in controls but did not rise markedly; in addition, there were no differences in expression among groups subjected to OGD for 4, 12, and 24 h (Figure 2B).

### Upregulated MiR-210-3p Enhances the Angiogenic Ability of EPCs Under Normal Oxygen Conditions

**FIGURE 3:**
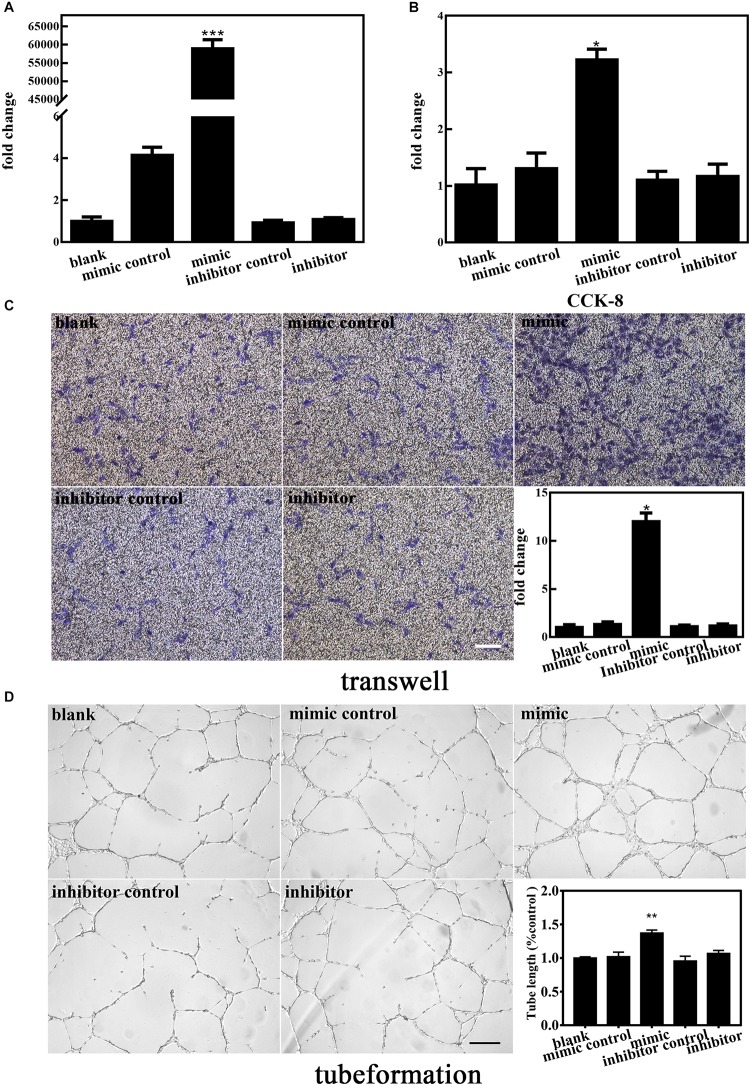
Effect of altered miR-210-3p expression on normal endothelial progenitor cell (EPC) angiogenesis. **(A)** Expression of miR-210-3p increased obviously in the mimic group. **(B)** Graph of CCK-8 results showed that the proliferation rate in the mimic group was improved compared to that in the other four groups. **(C)** Representative images showing EPC migration. Bar graph indicates that overexpression of miR-210-3p in EPCs resulted in enhanced migration ability. Bar = 100 μm. **(D)** Representative images showing EPC tube formation ability. Bar graph indicates that overexpression of miR-210-3p in EPCs resulted in larger tube length. Bar = 250 μm; *n* = 3 per group. Data are presented as the mean ± SEM, *p < 0.05, **p < 0.01, and ***p < 0.001.

To elucidate the involvement of miR-210-3p in the functional behaviors of EPCs, we transfected a miR-210-3p mimic, mimic control, miR-210-3p inhibitor, and inhibitor control into EPCs and subjected them to normal oxygen conditions. MiR-210-3p was upregulated in the mimic group but was not downregulated by the inhibitor (Figure 3A). We chose CCK-8, transwell, and Matrigel assays to test the proliferation (Figure 3B), migration (Figure 3C), and tube formation (Figure 3D) abilities, respectively, of these cells. Results showed that the EPC-miR-210-3p mimic group exhibited enhanced proliferative and migration abilities compared to EPCs alone. A similar outcome was observed for tube formation assays. Further, the EPC-miR-210-3p mimic group displayed the best tube formation ability among all groups. Other experimental conditions had no effect on EPCs. These data suggested that upregulated miR-210-3p expression enhances the angiogenic ability of EPCs under normal oxygen conditions.

### Upregulated MiR-210-3p Enhances the Angiogenic Ability of EPCs Under OGD Conditions

**FIGURE 4:**
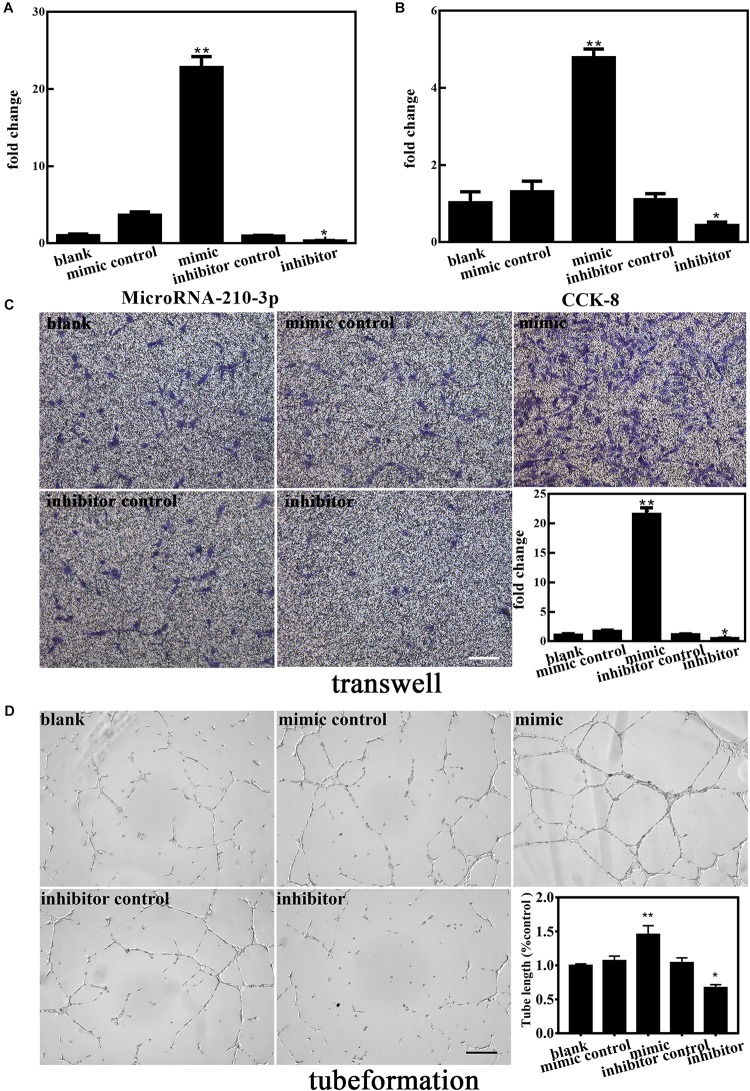
Effect of altered miR-210-3p expression on angiogenesis of endothelial progenitor cells (EPCs) under oxygen and glucose deprivation (OGD). **(A)** The expression of miR-210-3p increased in the mimic group but the opposite results were observed in the inhibitor group. **(B)** Graph of CCK-8 results showing that the proliferation rate in the mimic group was improved compared to that in the blank group, with opposite results in the inhibitor group. **(C)** Representative images showing the effects of 4 h of OGD on EPC migration. Bar graph indicating that the overexpression of miR-210-3p under OGD conditions for 4 h resulted in higher migration ability, with opposite results observed in the inhibitor group. Bar = 100 μm. **(D)** Representative images showing tube formation under the condition of OGD for 4 h in EPCs. Bar graph indicating that the overexpression of miR-210-3p resulted in EPCs resulted in larger tube length under the OGD condition, with opposite results observed in the inhibitor group. Bar = 100 μm; *n* = 3 per group. Data are presented as the mean ± SEM, *p < 0.05 and **p < 0.01.

To further elucidate the involvement of miR-210-3p in the functional behaviors of EPCs, we transfected a miR-210-3p mimic, mimic control, miR-210-3p inhibitor, and inhibitor control into EPCs under OGD conditions and performed the same experiments. MiR-210-3p was upregulated in the mimic group and downregulated in the inhibitor group (Figure 4A). We found that the EPC-miR-210-3p mimic group still exhibited enhanced proliferation, migration, and tube formation compared to those in EPCs alone under OGD conditions. However, the EPC-miR-210-3p inhibitor group showed the opposite result. Specifically, miR-210-3p inhibitor treatment resulted in reduced proliferation, subdued migration, and diminished tube formation (Figures 4B–D). These data suggested that upregulated miR-210-3p enhances the angiogenic ability of EPCs under OGD conditions, and in contrast, that downregulation of miR-210-3p inhibits these processes.

### MiR-210-3p Targets RGMA

**FIGURE 5:**
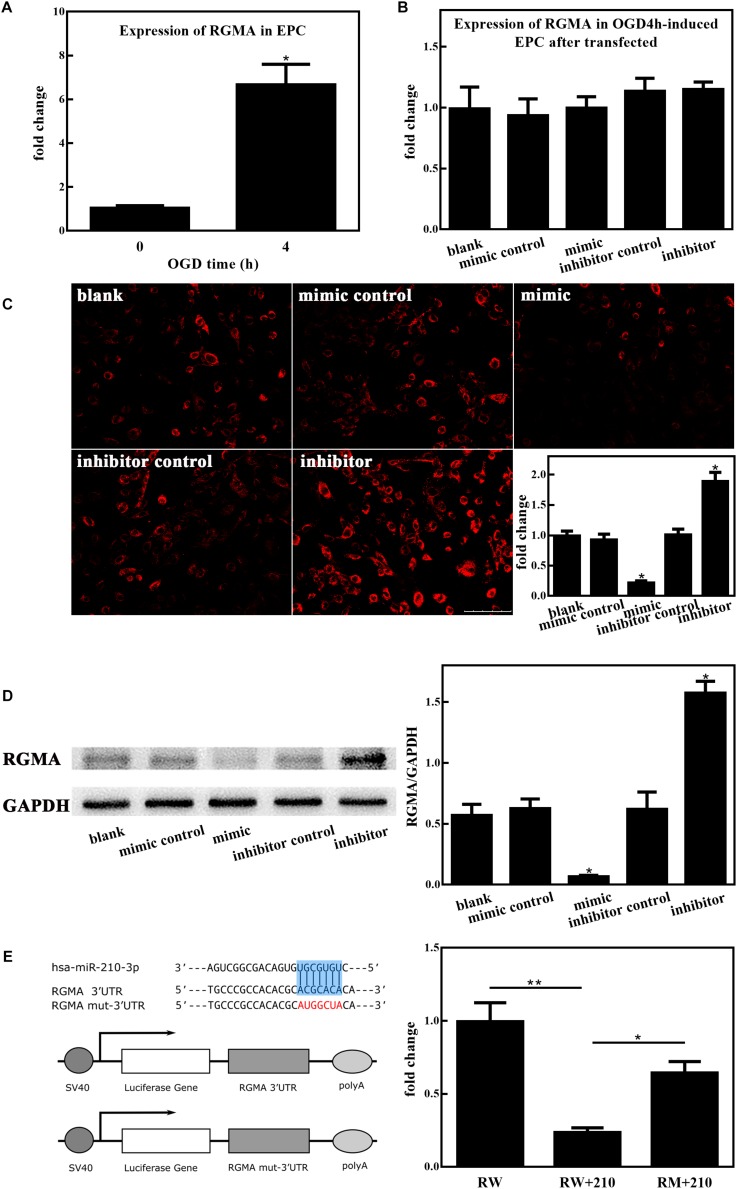
MiR-210-3p overexpression enhances angiogenesis by inhibiting RGMA. **(A)** Bar graph indicating that the expression of *RGMA* mRNA increased in endothelial progenitor cells (EPCs) upon culture under oxygen and glucose deprivation (OGD) conditions for 4 h. **(B)** There were no statistically significant differences among groups in terms of the expression of *RGMA* mRNA. **(C,D)** Immunohistochemistry staining and western blot results of EPCs cultured under conditions of OGD for 4 h after transfection; the expression of RGMA protein were reduced in the mimic group, and opposite results were observed in the inhibitor group. Bar = 100 μm. **(E)** Luciferase reporter assay showing that miR-210-3p can directly bind *RGMA*; *n* = 3 per group. Data are presented as the mean ± SEM, *p < 0.05 and **p < 0.01.

We next investigated the involvement of upregulated miR-210-3p, induced by OGD, in regulating RGMA expression in EPCs. First, the nucleotide coding region of *RGMA* mRNA was analyzed. A screen utilizing the online miRWalk software revealed putative binding between miR-210-3p and the *RGMA* 3′UTR. Further, *RGMA* mRNA expression was increased in EPCs alone under OGD conditions (Figure 5A) but there was no difference among groups after mimic or inhibitor of miR-210-3p was transfected into EPCs under OGD conditions (Figure 5B). Subsequent experiments showed that the protein expression of RGMA was significantly suppressed in the miR-210-3p mimic group of EPCs but increased in the miR-210-3p inhibitor group (Figures 5C,D and Supplementary Figure S1). In addition, when performing reported assays, co-transfection of RGMA-WT and the miR-210-3p mimic resulted in the significant downregulation of luciferase activity compared to that with transfection of RGMA-WT alone or with co-transfection of RGMA-MT and the miR-210-3p mimic. In addition, upon comparing RGMA-WT alone and RGMA-MT and miR-210-3p mimic co-transfection groups, no significant difference was noted, supporting the contention that the miR-210 mimic can directly bind the *RGMA* 3′UTR (Figure 5E). Hence, *RGMA* is a target of miR-210-3p.

## Discussion

The results presented in our study are the first to reveal that miR-210-3p is overexpressed in OGD-treated EPCs. Further, miR-210-3p overexpression might enhance the angiogenic ability of EPCs under hypoxia conditions via the suppression of RGMA expression at the protein level. This study could provide a theoretical basis to uncover the molecular mechanisms underlying recovery after stroke and suggest a new treatment approach for this event.

Previously, only the 3-arm of precursor microRNA (designated as the guide strand) was thought to mature and become functional, whereas the complementary 5′-arm (referred to as microRNA* or the passenger strand) was considered destined for degradation. However, research results in recent years have discovered that both arms of the pre-microRNA might be expressed differently and modulate various aspects of biological processes (Essandoh et al., 2016). To avoid confusion, miRNA/miRNA* nomenclature is now replaced by miRNA-3p and miRNA-5p, respectively. To this end, we explored the expression of both arms of miR-210, namely miR-210-3p and miR210-5p, in OGD-treated EPCs. MiR-210-3p is the guide-strand that integrates into the RNA-induced silencing complex, whereas miR-210-5p is the passenger-strand that is inactivated through degradation (Bartel, 2004). In this study, we examined the expression of miR-210-3p and miR-210-5p on EPC cells after OGD in the meantime. The results showed that the expression of miR-210-3p was significantly increased after hypoxia, which was several tens of times higher than the baseline level. And the expression of miR-210-3p was positively correlated with OGD time. However, Although 5p increased after ischemia, the expression of miR-210-5p was very low, only 3–5 times higher than baseline, and there was no noticeable correlation with time increase. Based on the above facts, we speculate that during the formation process of mature miRNAs (miR-210-3p and -5p) from precursor miR-210, miR-210-3p plays the major biological function. So for subsequent experiments on EPC function under hypoxic conditions, we focused on miR-210-3p to explore the underlying molecular mechanism.

With a functional hypoxia-responsive element in its promoter region (Huang et al., 2009), miR-210 has been found to be expressed at high levels in many cell types such as cardiomyocytes (Martinez et al., 2017), adipose-derived stem cells (Kim et al., 2013), and tumor cells (Ying et al., 2011; McCormick et al., 2013) upon exposure to hypoxia. Moreover, miR-210 has been shown to be related to the development of different diseases including immunological diseases (Wang et al., 2014), cancers (Ying et al., 2011; McCormick et al., 2013), and cardiovascular and cerebrovascular diseases (Zeng et al., 2016; Martinez et al., 2017). Accumulating evidence has shown the diverse functions of miR-210 in multiple critical biological processes concerned with hypoxia such as cell cycle, proliferation, angiogenesis, apoptosis, DNA damage repair, mitochondrial metabolism, differentiation, and tumor growth. Hypoxia decreases cell viability, migration and invasion while increasing apoptosis. Previous studies demonstrated that miR-210 is upregulated in the serum of patients with acute cerebral infarction and that it might be involved in the pathogenesis of this disease by regulating the proliferation and apoptosis of endothelial cells (Wang et al., 2018). Similar to these results, our study demonstrated that the overexpression of miR-210, and specifically miR-210-3p, is precise and protects EPCs against OGD injury, in addition to enhancing angiogenesis in OGD-treated EPCs. We also showed that a miR-210-3p mimic could ameliorate hypoxia-induced injury in EPCs, whereas a miR-210-3p inhibitor had the opposite effects. In addition, among all data, statistics showed that the expression of miR-210-3p could not be suppressed under normal oxygen conditions to affect cell function. We suggested that this might due to its low original expression such that the inhibitors could not function efficiently. However, inhibitors worked well and restrained the angiogenic ability of EPCs under OGD conditions, which confirmed our hypothesis. Based on these data, miR-210-3p might have a possible proangiogenic effect on EPCs.

Research indicates that EPCs can contribute to the growth of new blood vessels, potentially restoring the health of ischemic tissue (Tongers et al., 2010; Fadini et al., 2012). Upon searching ClinicalTrials.gov, many trials involving EPCs for the treatment of ischemia have used systemic infusion directly as the delivery method. However, most results showed poor efficacy due to high cell death and a lack of control with respect to cell targeting. EPCs are thus limited in their applications. One strategy to improve therapeutic outcomes is to combine such approaches with molecular therapy, thereby improving the survival rates and increasing the probability that cells will contribute to new blood vessel growth. We suggest that miR-210-3p overexpression could help to solve the existing problems and contribute to the application of EPCs to clinical treatment.

To shed light on the molecular mechanisms underlying the miR-210-3p-mediated control of cellular behavior, we used a bioinformatics method to virtually screen possible targets of miR-210-3p. Based on this, we focused on the interaction between miR-210-3p and *RGMA*, as results indicated that RGMA could be negatively regulated by miR-210-3p. Additional luciferase reporter assays further established that the *RGMA* 3′UTR was recognized by miR-210-3p. In previous studies, RGMA was shown to limit axonal regeneration after CNS injury (Hata et al., 2006) and to modulate T cell responses involved in autoimmune encephalomyelitis (Muramatsu et al., 2011). In summary, early research on RGMA was mainly focused on inflammation and autoimmune diseases. Recently, studies on the correlation between RGMA and ischemic disease have increased gradually. Zhang et al. found that RGMA suppresses the proliferation, tube formation, and migration of ECs by downregulating VEGF and p-FAK (Tyr397) via neogenin and Unc5b *in vitro* (Zhang et al., 2017). Wang et al. reported that RGMA might suppress angiogenesis via VEGF, Ang2, Ang1, and BDNF after cerebral ischemia/reperfusion injury through neogenin, thereby inhibiting neovascularization (Wang et al., 2018). Results of our study found that miR-210-3p overexpression has positive effects on cell proliferation, tube formation, and migration, suggesting that miR-210-3p might function through the negative regulation of RGMA. In addition, there was no difference in *RGMA* mRNA expression among groups, whereas the protein expression was significantly suppressed by a miR-210-3p mimic, suggesting that this miRNA primarily acts on gene translation and post-translational modification instead of transcription. Our study is the first to report that RGMA is a target of miR-210-3p in EPCs under hypoxia, and is consistent with previous reports (Wang et al., 2018) on the function of RGMA in ECs.

## Conclusion

In conclusion, we report that miR-210-3p protects EPCs against OGD injury and enhances angiogenesis in OGD-treated EPCs. The suppression of RGMA, directly regulated by miR-210-3p overexpression, might partially contribute to the protection against hypoxic injury in EPCs and enhanced angiogenesis. Considering the potential application of these results, there are still many unknowns that require further exploration. For example, it will be more stringent to perform an unbiased screen by mass proteomics to identify the preferred targets of this miR. Secondly, downstream signaling pathways, in addition to the interaction between miR-210-3p and RGMA, in OGD-treated EPCs need to be studied. Moreover, we also need to further design related experiments *in vivo* to investigate Whether miR-210-3p overexpression could enhance the angiogenic function of EPCs on an MCAO mice model and the post-stroke long-term functional recovery.

## Author Contributions

L-LZ involved in all aspects of the study, supervised the tissue assay analysis, and participated in the study design, data analysis, and revision of the manuscript. X-YX participated in the revision of the manuscript and data analysis. W-JL drafted the enclosed manuscript and collected the human donor cells. H-BL performed the data analysis. Y-FL, X-QT, and K-QD revised the manuscript. J-RH and G-YY participated in the design of the present study and revision of the manuscript.

## Conflict of Interest Statement

The authors declare that the research was conducted in the absence of any commercial or financial relationships that could be construed as a potential conflict of interest.
