# Supplementary figures and images for: MicroRNA-210-3p Targets RGMA to Enhance the Angiogenic Functions of Endothelial Progenitor Cells Under Hypoxic Conditions
Source: Front Cell Neurosci. 2019 May 21;13:223. doi: 10.3389/fncel.2019.00223 (PMC6536652; doi:10.3389/fncel.2019.00223)

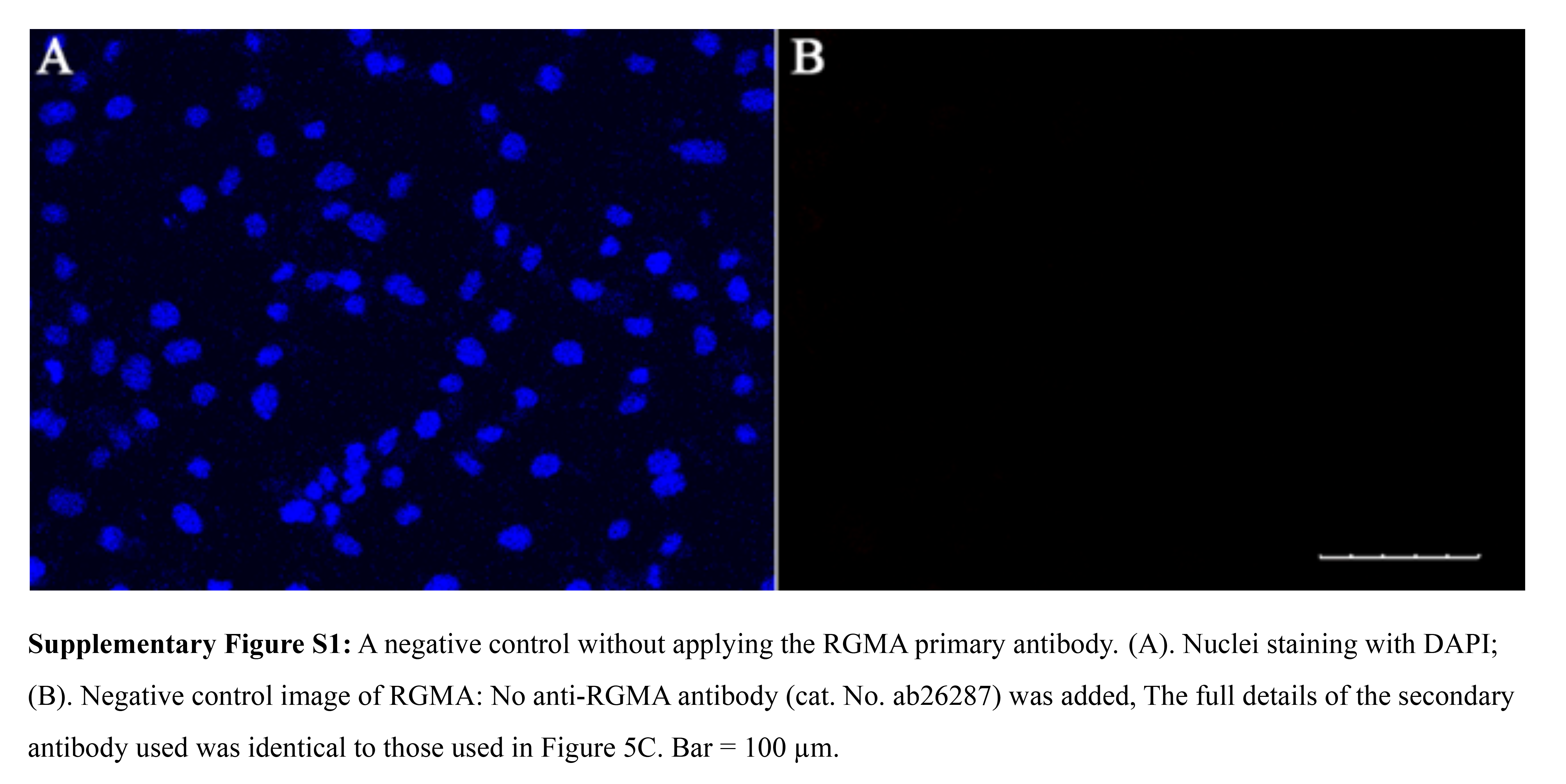

Supplement: Supplementary file 1 [file Image_1.tif]
